# Charge Your Brainzzz: the systematic development of a whole systems action program promoting sleep health in adolescents

**DOI:** 10.1186/s12889-025-23989-2

**Published:** 2025-10-17

**Authors:** Danique M. Heemskerk, Vincent Busch, Jessica T. Piotrowski, Carry M. Renders, Maartje M. van Stralen

**Affiliations:** 1 Department of Health Sciences, Faculty of Science and Amsterdam Public Health Research Institute, Vrije Universiteit Amsterdam, Van der Boechorststraat 7, 1081 BT Amsterdam, The Netherlands; 2 Department of Healthy Living, Sarphati Amsterdam, Public Health Service (GGD), 1018 WT Amsterdam, The Netherlands; 3 Amsterdam School of Communication Research ASCoR, University of Amsterdam, 1018 WV Amsterdam, The Netherlands

**Keywords:** Adolescent, Behavior change, Health behavior, Health promotion, Intervention development, Intervention mapping, Sleep, Systems science approach, Whole systems action program, Youth

## Abstract

**Background:**

Inadequate sleep among Dutch adolescents is a complex public health issue with detrimental effects on physical and mental well-being. Previous interventions have shown limited or no lasting effects. While systems approaches help to understand and address such challenges, effective prevention efforts require theory- and evidence-based intervention design using behavior change techniques. This study outlines the systematic development of a ‘whole systems action *program’*, named Charge Your Brainzzz (CYB), to promote healthy sleep in Dutch adolescents aged 12–15, by integrating theory- and evidence-based behavior change methods using the Intervention Mapping Protocol within a systems science approach, combined with stakeholder engagement.

**Methods:**

The CYB program was developed based on previously identified key system dynamics influencing adolescent sleep and a detailed action plan targeting these dynamics. The development was guided by a procedure of which several sub-steps from the Intervention Mapping protocol were applied: defining the program goal and prioritizing system dynamics outcomes across various interconnected subsystems, specifying performance objectives, constructing matrices of change objectives, developing program components while selecting theoretical methods and practical applications, and program production and formative testing. Adolescents, parents, teachers, school boards, school care coordinators, youth healthcare professionals, and Healthy School advisors were actively involved.

**Results:**

The theory- and evidence based whole systems action program CYB consists of eight program components and includes: 1) an educational component, 2) a step-by-step guide for implementing school sleep health policies, 3) a parent information evening, 4) an online magazine, 5) Teen Sleep Check, 6) Sleep Guide, 7) Tool for monitoring and early detection for sleep (behavior) problems, and 8) implementation materials including a website.

**Conclusions:**

The CYB program is the first whole systems action program designed to promote adolescent sleep health. Using a Systems Science approach, the Intervention Mapping (IM) protocol, and stakeholder engagement, the program addresses the complexity of the health issue, ensures a solid theoretical foundation for behavior change, and incorporates the lived experiences of the target groups. Beyond presenting the program’s components, this study offers a replicable roadmap for addressing complex public health challenges, paving the way for innovative, system-oriented solutions in health promotion.

**Supplementary Information:**

The online version contains supplementary material available at 10.1186/s12889-025-23989-2.

## Background

Poor adolescent sleep health is an increasingly recognized public health concern in many countries [1]. The Netherlands is no exception with over 50% of adolescents aged 14–17 sleeping less than the recommended minimum of 8 h per night [2] and 24% of adolescents aged 12–16 rating their sleep quality as poor [3]. This worrisome trend is further illustrated by the fact that the prevalence of sleep health problems among youngsters aged 12–25 is still increasing. In 2022, 22% percent of Dutch adolescents reported sleep problems compared to 14 percent in 2017 [4]. Inadequate sleep health is intricately linked to various aspects of adolescent physical health, mental health and cognitive performance, including for example physical inactivity, decreased emotion regulation, increased risk-seeking behaviors and poor academic performance [5]. The increasing prevalence of inadequate sleep health among adolescents and its adverse impacts on adolescent life emphasizes the need for interventions addressing sleep health in this population.

Thus far, preventive interventions aimed at promoting healthy adolescent sleep, via e.g., sleep education or relaxation techniques, have shown only minor or no significant, lasting effects [6]. A factor that likely hampers the effectiveness of current preventive adolescent sleep health interventions is that most have not taken into account the complexity of sleep health in the intervention design [7, 8]. Instead, existing interventions tend to target specific social cognitive determinants, such as knowledge or attitudes, within specific settings like schools [8]. However, adolescent sleep health is far more complex, being shaped by the dynamic interplay between many biological, economic-, physical-, sociocultural-, and political factors. In as much, there is a clear need for a comprehensive whole systems approach that embraces and integrates this complexity [9].

Systems thinking methodologies can be applied to gain a good understanding of the complex health problem at hand, as well as to develop comprehensive approaches to tackle it [10]. A recent study illustrated this by providing insights into the determinants of adolescent sleep health, the system dynamics (e.g., interrelations and feedback loops), and the underlying mechanisms that integrate them into a holistic complex system. It also identified potentially impactful leverage points to focus sleep health promotion efforts on in order to realize effective, durable system changes [9]. Subsequently, another study showed how such insights were used to develop a ‘whole systems action *plan’*, which outlines a wide range of proposed actions, serving as a guide for strategic planning and implementation of initiatives aimed at driving systemic changes [11]. Both studies make clear that no single action or stand-alone intervention can bring about the significant and lasting effects to meaningfully affect adolescent sleep health, but that it will take a comprehensive collection of efforts to do so.

However, alongside taking this complexity into account, any successful effort to durably and effectively impact adolescent sleep health should consist of more than a collection of potential actions; such actions should be shaped using theory- and evidence-based intervention design techniques, apply appropriate behavior change techniques, and form one coherent whole rather than a collection of parts [6, 11]. The previously described whole systems action *plan* should thus be shaped into a coherent, theory- and evidence-based whole systems *program* (i.e., intervention)*.* One extensively used approach for developing theory- and evidence-based health promotion programs is the Intervention Mapping (IM) Protocol [12]. IM provides a systematic iterative framework that integrates theoretical knowledge (e.g., appropriate theoretical methods) and scientific evidence *with* actor participation throughout the developmental process of a health promotion program. A key feature of the IM Protocol is that it guides the selection of appropriate, theory-based behavior change methods to shape health promotion programs [13]. It provides a structured approach to choose strategies and techniques (i.e., behavior change methods), based on theoretical and empirical evidence, to influence specific behavioral determinants. As a result, the intervention has a strong theoretical foundation and its potential impact and sustainability are optimized.

Systems thinking provides a holistic perspective on health problems, by identifying dynamic interactions between factors across multiple levels, revealing potential leverage points, and guiding actions on several system levels to initiate system change. Intervention Mapping, in turn, provides a strong theoretical foundation for behavioral change methods, ensuring that actions/intervention strategies are theoretically grounded, enhancing their effectiveness. Together, these approaches enable the development of interventions that are both evidence-informed and responsive to the complexity of real-world systems. To the best of the authors knowledge, no whole systems action program exists that applies a systems perspective to address the complexity of a health problem and integrates theory- and evidence-based behavior change methods through a structured development process like the Intervention Mapping Protocol.

To address this notable gap, the objective of the current study is twofold. First, we aim to systematically develop and describe a ‘whole systems action program’ to promote healthy sleep among Dutch adolescents aged 12–15, by integrating theory- and evidence-based behavior change methods through the Intervention Mapping Protocol. Second, we aim to provide a detailed account of how Intervention Mapping can be meaningfully embedded within a Systems Science approach, offering practical guidance for both systems scientists and intervention developers seeking to design health promotion programs that are both theoretically robust and contextually grounded.

## Methods

### Design

**Fig. 1:**
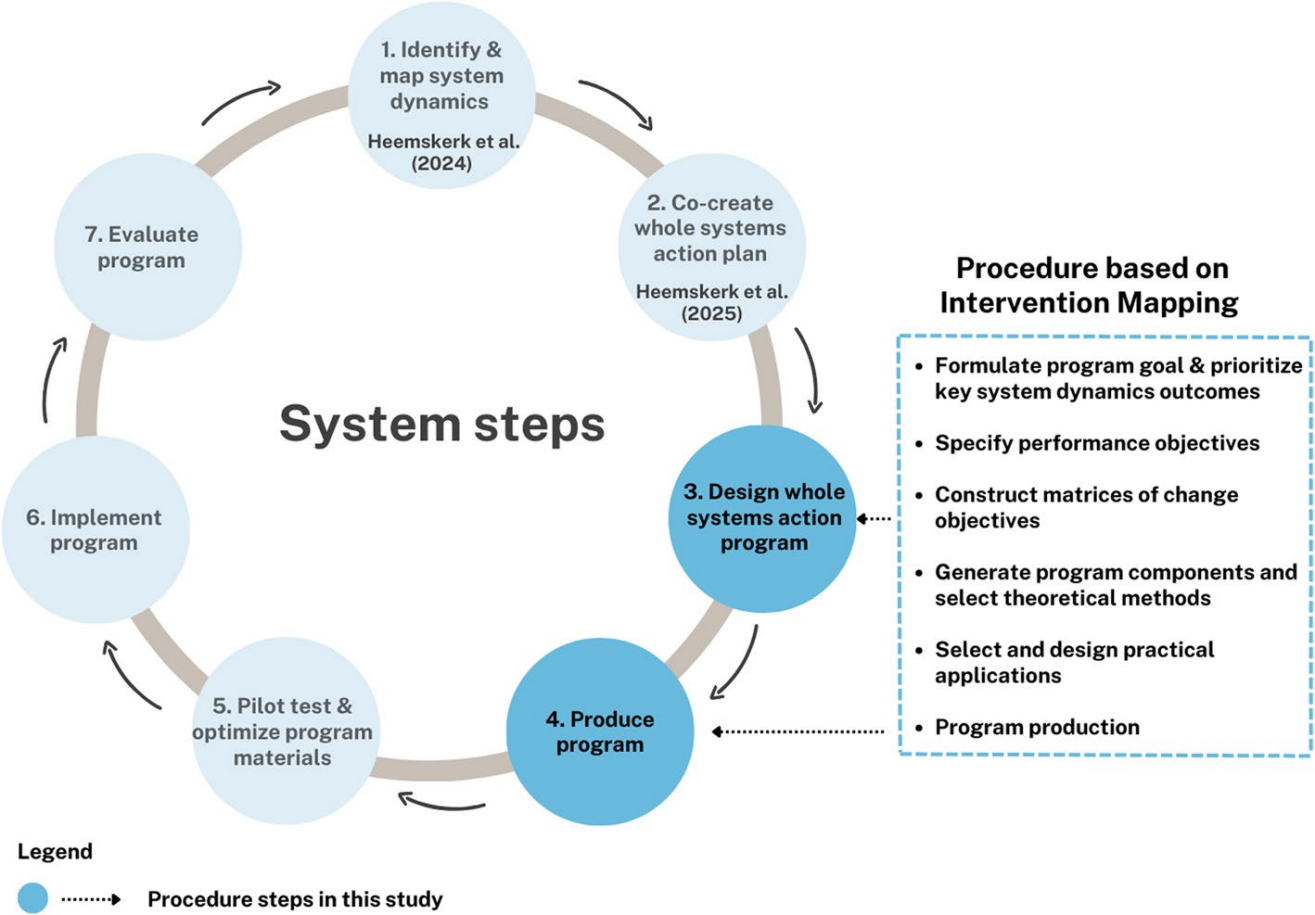
System Steps to develop a whole systems action program

The current paper presents the development and details of a whole systems action program, named the ‘Charge Your Brainzzz (CYB) program’, to promote the sleep health of Dutch adolescents aged 12–15. Figure 1 provides an overview of the ‘system steps’ followed in our comprehensive systems approach to create the program. Step 1 and 2 were conducted in previous studies, yet were visualized here as well to make clear all the steps that laid the foundation for the current study. The current study specifically outlines steps 3 and 4, illustrating the development process of the theory- and evidence-based whole systems action program. Prior to study onset, ethical approval was granted by the institutional medical ethics committee of Amsterdam UMC (VUMC 2021.0783).

### Procedures

**Fig. 2:**
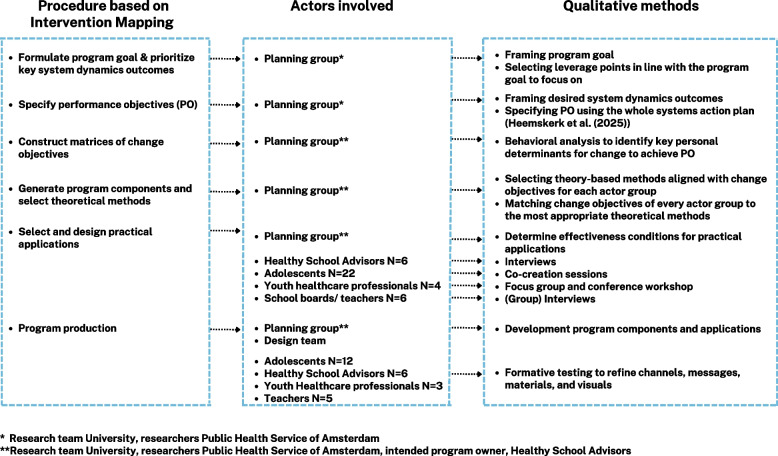
Overview procedure and actors involved

To develop the whole systems action program, we integrated selected sub-steps of the Intervention Mapping Protocol – specifically those related to the selection and integration of behavior change methods (step 2 & 3 of the IM protocol) – within system steps 3 and 4, thereby ensuring a strong theoretical foundation for behavior change (Fig. 1). This integration required adaptations to several IM steps in both earlier work [9, 11] and the present study. Specifically, IM Step 1 (the logic model of change) was adapted to incorporate a systems perspective by embedding a previously developed causal loop diagram [9] and action plan [11] into the needs assessment. Furthermore, we replaced the ‘behavioral outcomes’ and ‘environmental outcomes’ columns in the IM logic model with ‘system dynamics outcomes’ to better reflect key leverage points and the interplay of behavioral and environmental mechanisms affecting adolescent sleep health. The next two steps in Fig. 1, steps 3 and 4, make up the current study in which we defined performance objectives and constructed matrices of change objectives (IM Step 2), then selected theoretical methods and practical strategies to inform program components (IM Step 3), and finally developed the intervention materials (IM Step 4). Figure 2 visualizes which actors were involved at each stage and how they contributed. Detailed descriptions of these procedural steps are described in more detail below.

#### Formulate program goal & prioritize key system dynamics outcomes

The planning group started by formulating a program goal (Fig. 2). In addition, the group prioritized *key system dynamics outcomes* (i.e., leverage points) that the CYB program should focus on to effectively and sustainably improve adolescent sleep health. As the selected outcomes aligned with the Healthy School Approach (see Table 1), which is a preventive infrastructure aimed at promoting health among adolescents in the Netherlands, all consecutive steps were conducted in accordance with this infrastructure.

**Table 1:** The Healthy School Approach

In the Netherlands, the Healthy School approach provides a central public health infrastructure to implement health promotion initiatives [14]. The approach is funded by the Dutch government and is nationally coordinated by the Association of Public Health Services (GGD GHOR Nederland), the National Institute for Public Health and the Environment (RIVM), and the primary, secondary, and vocational education councils (in Dutch: PO-Raad, VO-Raad, and MBO Raad). The Dutch Healthy School Approach provides a structured yet flexible framework for integrating health promotion activities within Dutch primary and secondary schools. Based on the WHO’s Health Promoting School Model [15], it focuses on targeting four ‘pillars’ per health topic to achieve health behavior changes, i.e., (1) health education, (2) a healthy social and physical school environment, (3) healthy school policies, and (4) monitoring and early detection of specific health or health behavior issues among children that warrant special attention or care of other health professionals [14]. Sleep health was recently adopted as one of the ten core Healthy School topics with no intervention program(s) available to address it. Given this and the fact that several impactful causal mechanisms and leverage points to influence adolescent sleep health involve the school setting [9], we aimed to align the CYB program with the Healthy School framework. This will also facilitate future implementation of the program.	

#### Specify performance objectives

As a next step and based on the prioritization of the key system dynamics that the CYB program should focus on (e.g., adolescents do their homework in the evening), the planning group defined the *desired system dynamics outcomes* (e.g., ‘adolescents limit doing homework in the evening’). From that, we specified *performance objectives* for all actors contributing to the desired system dynamics outcomes (i.e., adolescents, parents, teachers, school boards, school care coordinators, youth healthcare professionals and Healthy School advisors) in accordance with the IM Protocol [12] (Fig. 2). A performance objective describes a specific, observable action or behavior that an actor needs to perform to contribute to a desired outcome. Performance objectives are measurable and are directly linked to the desired outcome. For example, the performance objective for teachers might be: *‘avoid assigning homework for the following day,’* contributing to the broader goal or desired system dynamic outcome: *‘adolescents limit doing evening homework’,* thereby positively impacting their sleep health. As input for specifying CYB’s performance objectives, we used the previously developed ‘whole systems action plan’ by Heemskerk et al. [9], which outlined an extensive list of actions that could be taken to change the system dynamics. The complete set of performance objectives outlines all the specific actions that all actors involved could take to achieve the program goal.

#### Construct matrices of change objectives

Subsequently the planning group formulated *change objectives* which specify what needs to change in terms of behavioral determinants (e.g., knowledge, attitudes, beliefs or abilities) for all actors to successfully perform the desired performance objectives. While performance objectives outline *what* needs to be done, change objectives focus on *how* to achieve that desired behavior by addressing the underlying personal determinants that influence behavior. For example, in the CYB program, a performance objective for teachers is: *‘avoid assigning homework for the following day’*. To support this behavior, change objectives could include that it is necessary to increase teachers’ *knowledge* about the negative effects of evening homework on adolescents’ sleep health, to raise *awareness* of their own influence regarding assigning/communicating homework not on time, and to identify *barriers* for not assigning/communicating homework for the next day on time.

These change objectives were organized into matrices to outline the necessary changes in personal determinants required to achieve all performance objectives among all included actors and to achieve the program goals of CYB. Relevant determinants were identified using behavioral models such as the Social Cognitive Theory [16] and Theory of Planned Behavior [17]. Key determinants included: knowledge, awareness, attitude/beliefs, self-efficacy/barriers, subjective norms and skills.

#### Generate program components and select theoretical methods

Next, the planning group brainstormed program themes and components tailored to each actor group for which performance objectives were developed (i.e., adolescents, parents, teachers, school boards, school care coordinators, youth healthcare professionals and Healthy School advisors) (Fig. 2). Thereafter, the research team selected theoretical methods that align with the specified change objectives identified [13]. Theoretical- and evidence based behavior change methods refer to general techniques grounded in behavioral or psychological theories and have been found to be effective in influencing behavioral determinants. We used theories such as Theory of Planned Behavior [17], Social Cognitive Theory [16], Health Belief Model [18], and Theory of Goal-Directed Behavior [19]. For example, ‘modeling’ is a method derived from the Social Cognitive Theory that can be applied to change a social norm. Each change objective for every actor group was matched to the most appropriate theoretical methods.

#### Select and design practical applications

Thereafter, the planning group translated these methods into practical strategies or applications (Fig. 2). For instance, the abstract theoretical concept ‘modeling’ can be translated into a practical application such as creating a video that showcases relatable individuals successfully performing a target behavior. The choice of application depends on the personal determinant(s) that need to be influenced as well as its/their ‘conditions for effectiveness’. These conditions are crucial since theoretical methods are not universally effective; they typically require certain parameters to be met. In the case of ‘modeling’ for example, it is vital to provide an appropriate, i.e., relatable, model performing a desired action or behavior [13].

To further ensure that the program components and practical applications were not only theoretically appropriate but also aligned with lived experiences of the target group, active involvement from relevant actor groups was prioritized. This process of co-designing ensured the applications were relevant, feasible and acceptable for implementation. First, to ensure that the program components and practical applications align with the Dutch prevention infrastructure for promoting health in schools, input was gathered from Healthy School Advisors (*N* = 6). These advisors, who support and guide schools in implementing health-promoting practices, policies, and processes, were asked about their specific needs and requirements (See Additional File 1 for interview structure) [20]. Interview notes were summarized and thematically analysed. Healthy School Advisors were recruited via the network of the consortium’s affiliated municipalities and regional Public Health Services.

Second, to design practical applications that aligned with the lived experience of adolescents, we conducted two co-creation sessions with adolescents (*N* = 22). These sessions aimed to explore how adolescents prefer to learn about sleep and to identify suitable practical applications (see Additional File 2 for description of the co-creation structure developed for this study). We selected adolescents from pre-vocational secondary education (in Dutch: VMBO^1^) [21] aged 12–15 years, as this group experiences the poorest sleep health amongst Dutch adolescents [3]. Therefore, these teenagers were the focus for the CYB program. Notes, post-its and other used materials were summarized and thematically analyzed. Adolescents were recruited through schools via our Charge Your Brainzzz consortium network and via a youth panel (containing over 7500 members between the ages of 12 and 24). Participating youth received a gift card of 25 euros and participating classes received a class outing worth 250 euros.

Third, to identify both potential suitable components and practical applications for the school care coordinator – a professional within a school setting responsible for ensuring that students with diverse needs (e.g., academic, social, emotional, or physical) receive appropriate care and interventions – and youth health professionals, a focus group was held (*N* = 4) and additional perspectives of this target group were gathered during a conference for youth healthcare professionals (see Additional File 3 for description of the focus group developed for this study). Notes, post-its and other used materials were summarized and thematically analyzed.

Fourth, to inform a policy advice for *school boards* to create a healthy school environment focused on sleep, the research team held (*N* = 6) interviews with school boards and/or teachers about potential implementable school policies (i.e., delaying school starting times, adapting communicating strategies and adjusting exam schedules) and the barriers and facilitators of implementing them. For data analysis, recordings of the interviews were transcribed and analysed in MAXQDA Qualitative Data Analysis Software version 2018.2, via an inductive thematic approach using open coding. The interviews held are part of another study and results can be found elsewhere [22]. School professionals and youth health professionals were recruited via LinkedIn, schools and the network of the consortium’s affiliated municipalities and regional Public Health Services.

While co-design enabled us to tailor the intervention to the lived experiences and preferences of the target population, it also demanded ongoing care to respectfully incorporate input from all stakeholders and ensure that selected ideas were both appropriate and evidence-based for the intended goals and context. Ideas proposed during co-creation sessions that lacked support from behavioral theory, existing evidence, or were deemed infeasible were not included in the final program. When such tensions arose, we engaged participants in open dialogue to transparently address these issues and collaboratively identify alternative solutions.

#### Program production & formative testing

Next, the designed program components and applications led to the actual program production and pretesting. All materials were co-produced and/or formative tested with the planning group, graphical designers, and with those involved with the components, either as recipients (adolescents *N* = 12) and/or as implementers (e.g., Healthy School Advisors *N* = 6, youth healthcare professionals *N* = 3, teachers *N* = 5) to ensure their appropriateness and acceptability. This included considerations such as communication channels, methods, messages, materials, protocols, as well as evaluating the program’s appeal, visual design, content, and the clarity of all materials and assignments.

## Results

### Formulate program goal & prioritize key system dynamics outcomes

The program goal of Charge Your Brainzzz is to improve the system to enhance the sleep health of adolescents from pre-vocational education aged 12–15 years. Several key system dynamics outcomes (i.e., leverage points) across the subsystems identified by Heemskerk et al. [9] – i.e., school environment, digital environment, mental wellbeing, family environment, and personal system – were prioritized as targets for the CYB program. Outcomes included, for example, early school starting times, evening homework, evening school notifications, parenting sleep practices, and evening screen use. An example of how we used the previously identified feedback loops and leverage points from the subsystem ‘school environment’ to form the system dynamics outcomes can be found in Additional File 4. While all subsystems were approached this way, to facilitate manuscript readability, we only report on the school environment as an example.

### Specify performance objectives

Table 2 specifies the desired system dynamics outcomes and 33 identified performance objectives of the subsystem ‘school environment’. For instance, to address the outcome of ‘*school schedules are aligned with adolescents’ biorhythm’*, a number of performance objectives were formulated for necessary changes to this mechanism. These include, for example, ‘*schools implement a policy that states that school start times are no earlier than 9 AM’* or ‘s*chools are provided with tools and guidance (e.g., policy templates) on how to implement changes to their school starting times, end times and exam timetables that allow for a better alignment with students’ biorhythm’*.

**Table 2:** Desired Ssystem Dynamics Outcomes and Performance Objectives – School environment subsystem

Desired system dynamics outcomes: School environment	Performance objectives*
• Adolescents limit doing evening homework • Adolescents do not experience difficulties planning their homework • Adolescents perceive less performance pressure fromschool	• Adolescents plan (with help of their parents) their daily activities in such a way to prevent homework in the evening • Teachers avoid assigning homework for the following day • Teachers communicate homework details in time, at the latest at 4:00 PM • Schools have a policy stating to assign little to no homework for the next day • Schools create a structure to provide support and guidance to adolescents in completing homework on time • Schools aim to provide support and guidance to adolescents in completing homework on time • Schools become aware that managing and planning homework can be particularly challenging for teenagers, given their developing organizational skills and competing priorities
• Late-night school deadlines are prohibited	• Teachers avoid setting late-night school deadlines • Schools implement a policy that restricts late-night school deadlines • Schools become aware that late-night school deadlines damage students’ sleep health, which in turn is crucial for their cognitive development, mental wellbeing and physical health as well as damaging their right to leisure time after school hours to keep a healthy school-private life balance and protect their mental wellbeing
• Homework is coordinated between teachers about when and how much homework they give	• Teachers coordinate homework assignments and exams with other subjects to prevent workload peaks • Schools schedule no exam weeks, but rather distribute exams evenly throughout each trimester to prevent peaks in workload and subsequent stress with students • Schools utilize one digital platform for communication and sharing homework assignments with adolescents to prevent unexpected workload peaks
• School schedules are aligned with adolescents’ biorhythm (e.g. starting- and end times, breaks)	• Schools implement a policy that states that they align school schedules with adolescents’ biorhythm (e.g., start times and end times, breaks) • Schools implement a policy that tests or examinations do not take place before 11:00 AM • Schools aim to align school schedules with students’ biorhythm • Schools become aware of the impact of school start time on sleep and cognitive development of adolescents, and the importance of better aligning school start times with adolescent biorhythm
• Schools do not digitally communicate about e.g., homework in the evening	• Teachers do not specify the exact day and time that they will publish students'test scores and grade • Teachers do not digitally communicate in the evening • Teachers become aware about the effect of evening communication and specifying the exact day and timing of grade publication on sleep health to strengthen the belief that this anticipation causes stress and in turn affects sleep health • Schools implement a policy that prohibits teachers from specifying when test grades will be published • Schools set their digital (mobile) applications to limit the accessibility of students to the online grading and test scores system between 8:00 PM and 8:00 AM • Schools implement policies that prevent sending students notifications as well as to prevent them from accessing the online test scores and grading system between 8:00 PM and 8:00 AM • Schools only use digital (mobile) applications that allow for sending students messages at preset times • Schools become aware that evening notifications (e.g. communicating test results) causes students stress that in turn negatively affects their sleep health • Schools become aware that their students have the right to leisure time after school hours to keep a healthy school-private life balance and protect their mental wellbeing
• Sleep health and teaching relaxing sleep hygiene practices are part of the school curriculum • Teachers’/school boards have a lack of awareness of students sleep health (or mental health) condition • Schools focus on promoting adolescent (sleep) health besides proving optimal cognitive, educational outcomes • Schools are there to facilitate learning and getting good grades and adolescent (sleep) health • Schools belief and are aware that they have a large responsibility in stimulating adolescent sleep health	• Adolescents learn about sleep health in school with the aim to stimulate their healthy sleep habits • Schools integrate sleep health intervention efforts into their curriculum • Schools become aware of their students’ sleep health condition • Schools monitor students’ sleep health condition regularly • Schools actively protect and stimulate the sleep health of their students • Schools prioritize overall well-being alongside academic achievement • Schools become aware that adolescent sleep health is crucial to healthy adolescent development, cognitive functioning and learning

^*^In the research conducted by Heemskerk et al., [11] actions were identified across various system levels using the Action Scales Model (i.e., event level, structure level, goal level, belief level) Nobles et al. [23]. These actions served as input for defining the performance objectives. For clarity reasons, the categorization of the performance objective across the ASM levels has been omitted

### Construct matrices of change objectives

Table 3 provides examples of the change objectives developed to align with the first seven performance objectives from Table 2. It outlines for all actors how specific personal determinants should be changed to achieve their specific performance objective(s). A comprehensive overview of all performance objectives and change objectives related to the subsystem ‘school environment’ can be found in Additional File 5.

**Table 3:** Performance Objectives and Change Objectives—School environment

School environment	Change objectives					
**Performance Objectives**	**Knowledge**	**Awareness**	**Attitude/beliefs**	**Self-efficacy/barriers**	**Subjective norms**	**Skills**
Adolescents plan (with help of their parents) their daily activities in such a way to prevent homework in the evening	Adolescents know and recognize the negative influences of evening homework on their sleep Parents know the negative influences of evening homework on the sleep of adolescents	Adolescents become aware of their activities during the day and opportunities for better planning Parents become aware of the perceived difficulties of adolescents with planning daily activities (including homework)	N/A Parents belief that adolescents at this age range cannot always take their own responsibility and still need a guiding role from their parents	Adolescents identify barriers for not finishing homework in the afternoon Express confidence in planning their daily activities in such a way to prevent homework in the evening Parents feel confident in providing help to their child with planning daily activities (including homework)	N/A Parents recognize that other parents also provide help with planning daily activities with their child	Demonstrate a plan of how to make an efficient planning of daily activities preventing evening homework N/A
Teachers avoid assigning homework for the following day Teachers communicate homework details in time, at the latest at 4:00 PM	Teachers know and recognize the negative effects of evening homework on adolescents’ sleep health	Become aware of their influence regarding assigning/communicating homework not on time on adolescent sleep	Believe that that their students have the right to leisure time after school hours to keep a healthy school-private life balance and protect their mental wellbeing	Identify barriers for not assigning/communicating homework for the next day/on time Express confidence in not assigning/communicating homework for the next day/on time	N/A	N/A
Schools have a policy stating to assign little to no homework for the next day Schools create a structure to provide support and guidance to adolescents in completing homework on time Schools aim to provide support and guidance to adolescents in completing homework on time Schools become aware that their students have the right to leisure time after school hours to keep a healthy school-private life balance and protect their mental wellbeing	Schools know and recognize the benefits of diminishing evening homework on adolescents’ sleep health and providing support and guidance in completing homework on time	Become aware of their influence regarding assigning/communicating homework not on time and providing support and guidance in completing homework on time on adolescent sleep	Believe that that their students have the right to leisure time after school hours to keep a healthy school-private life balance and protect their mental wellbeing	Identify barriers for not developing and implementing a policy regarding not assigning/communicating homework for the next day/on time Identify barriers for not providing guidance and support for students to complete homework on time Express confidence in developing and implementing a policy/agreement regarding not assigning/communicating homework for the next day/on time and setting up a structure to support and guide students in completing homework on time	Recognize that other schools also aim to prevent evening homework and provide support and guidance for students to complete homework on time	Demonstrate the ability to implement a policy/agreement that little to no homework is assigned for the next day Demonstrate the ability to provide support and guidance in completing homework on time

### Generate program components and select theoretical methods and practical program applications

The practical program components focused on health education, school policies, social and physical environment, and monitoring and screening aligning with the Healthy School infrastructure. As noted earlier, adolescents, parents, teachers, care coordinators, school boards, and Healthy School Advisors were identified as key groups to involve and would serve as CYB’s target audience and implementers.

Change objectives were matched to appropriate theoretical methods and thereafter translated into appropriate practical applications. For example, for the change objective that *adolescents should not take their phone to bed* the theoretical methods that were selected included: ‘planning coping responses’ (based on Relapse Prevention Theory), and ‘implementation intentions’ (based on Theories of Goal-Directed Behavior). The practical applications we selected include individual and class challenges that focus on goal setting and the development of coping strategies. These strategies are then collectively reflected upon in class to reinforce learning and commitment (Table 4).

**Table 4:** Overview of the Charge Your Brainzzz program components, theoretical methods and practical applications

Program components	Theoretical Methods	Practical applications/Tools
Healty School Pillar: Health education		
1. Charge Your Brainzzz – educational program An education component, consisting of four complementary lessons for first-year pre-vocational secondary school students aimed at conveying the importance of sleep, raising awareness regarding the significance of sleep, eliciting the causes of poor sleep, equipping students with strategies to improve their own sleep and sleep hygiene practices, and reflecting on first attempts to improve sleep and sleep hygiene practices	• Belief selection • Persuasive communication • Active learning • Individualization • Modeling • Feedback • Reinforcement • Facilitation • Nudging • Chunking • Discussion • Providing cues • Consciousness raising • Framing • Counterconditioning • Implementation intentions • Early commitment • Public commitment • Environmental reevaluation • Arguments • Direct experience • Resistance to social pressure • Guided practice • Self-monitoring of behavior • Provide contingent rewards • Cue altering • Goal setting • Planning coping responses • Entertainment education	The lessons utilize interactive methods. Each lesson started with a video with role models that introduced the topic of the lesson and served as a conversation starter. All lessons ended with a"challenge"with their classmates, teacher and/or parents wherein they made an action plan on reducing screen use, doing relaxation activities before sleep and on sleep hygiene practices, For example, after the lesson on screen use, the students committed to a class challenge, the T-day Challenge, to encourage adolescents to leave their phones outside their bedrooms on days that start with a T (i.e., Tuesdays and Thursdays) to reduce evening screen use (based on the study [24] Lesson 1: “Charge Your Sleep” focuses on becoming aware of their personal sleep schedules and the importance of sleep. It includes interactive activities such as a sleep mask quiz with yes/no statements about the importance of sleep and the consequences of inadequate sleep, fact or fiction statements, exercises within a workbook, and a “Charge Your Sleep- challenge”, where students set a specific goal to improve their sleep. They identify their intention, they set a sleep goal and plan when and how they will achieve this goal, and who can support them Lesson 2: “Charge Your JOMO” (i.e., Joy of Missing Out) focuses on the impact of evening screen use and social media on sleep. The lesson starts with a reflection on the previous challenge, with questions such as: *“Did you complete your challenge from the previous lesson? What went well? What did you still find difficult? How will you address this next time?”*. Students learn about how social media and screen use can influence sleep and participate in a dilemma game where they choose between two options that indirectly discourage screen use while providing alternatives for screen-free activities. The lesson also includes a video, workbook exercises and introduces the “T-Day Challenge,” where students set a specific goal to decrease their evening screen use Lesson 3: “Charge Your Chill Skills” focuses on the impact of stress and mental wellbeing on sleep. The lesson starts with a reflection on the T-Day challenge and a video. Students then complete a “stress meter” in their workbook to become aware of how stressed they feel and identify their stress triggers. Next, they reflect on activities that help them relax, learn the “military sleep method” to reduce overthinking and fall asleep more quickly, and create a campaign for a “chill advertisement”. The lesson ends with the introduction of the “Me-Day Challenge”, where students are challenged to try out various relaxation activities to find what works best for them Lesson 4: “Charge Your Dreams” focuses on reviewing and summarizing the material from previous lessons, helping students to identify practical tips to improve their sleep, and strategies to maintain these habits over time. The lesson begins with a reflection on the Me-Day Challenge and includes a video where students analyze the sleep hygiene of a role model, identifying what the role model is doing well and what needs improvement. Students then participate in a “Survival of the Fittest” quiz puzzle to test their knowledge of sleep facts, followed by a fact-or-fiction quiz. The lesson ends with a Sleep Challenge, where students select two personalized challenges to further enhance their sleep habits
Healthy School Pillar: School policies		
2. Step-by-step guide for forming and implementing school sleep health policies A step-by-step guide for Healthy School Advisors and schools to assist and inform school boards in formulating and implementing school sleep health policies. This guide aims to enhance self-efficacy and foster positive beliefs about the benefits and feasibility on the policy measures	• Belief selection • Modeling • Consciousness raising • Technical assistance	The guide includes insights and experiences form other schools, detailing how they successfully implemented specific policy measures and their outcomes. The step-by-step guide includes policies with regard to: • (Digital) Communication strategies; e.g., not sending students late-night messages regarding schedules, test results and other school-related information; • Adjusting test schedules; ensuring that the school does not schedule tests in the early morning hours; • Modifying school start times; ensuring that students start times are aligned with their age i.e., biorhythm In addition to offering guides with practical strategies for adopting, implementing, and sustaining these policy measures, the CYB-team provides a support and implementation dialogue on how these policy measures can be implemented within the school. It also includes general information about the importance of the policy measures, a FAQ, and shared experiences from other schools that have successfully implemented them
Healthy School Pillar: Social and physical environment		
3. A parent information evening The parent information evening aims to raise awareness about the importance of sleep and to provide parents with information about how to improve their teenagers’ sleep health. Topics covered include: the importance of good sleep, how to establish agreements with your teenager about bedtimes and screen use, and what the recommended bedtimes are for this age group 4. An online magazine The E-zine is a digital magazine that serves as a reference guide for parents. It offers advice and practical tips to help parents and caregivers guide their teenagers towards better sleep	• Belief selection • Modeling • Chunking • Discussion • Consciousness raising • Information about others’ approval	An engaging and interactive parent (or caregiver) information session to be held at school, focusing on key topics such as teenage brain development, the biological mechanisms of sleep, and parenting sleep practices to support healthy adolescent sleep. It lasts about an hour and provides parents with insights and practical tips on positively influencing their teen’s sleep, stress, and screen use. The presentation is supported by videos of experts on sleep and on parenting, a video with a role model play on making agreements, fact-or-fiction quiz and discussions within the group. The E-zine is a digital magazine that can be found on the website of Charge Your Brainzzz covering the same information as the parent information evening supplemented with references to the ‘Teen Sleep Guide’ and the ‘Teen Sleep Check’
5. The ‘Teen Sleep Check’ (Dutch: SlaapCheck Tiener) An online tool for parents to become aware of their teenager’s sleep health and sleep hygiene practices and to acquire knowledge about how to improve their teenagers’ sleep hygiene practices 6. The ‘Teen Sleep Guide’ (Dutch: Slaapwijzzzer) An online tool for parents to acquire knowledge about healthy sleep hygiene practices during the day	• Belief selection • Persuasive communication • Individualization • Chunking • Consciousness raising • Framing	Based on their response on a series of questions part of the Teen Sleep Check, parents receive an advice to improve their teenagers’ sleep, such as creating a good sleep environment, no screens within the bedroom, and emphasizing the importance of a consistent sleep routine The Sleep Guide offers practical tips on healthy sleep hygiene practices during daytime that parents can promote amongst their teenager to ensure a better night's sleep
Monitoring and screening		
7. Tool for monitoring sleep health and early detection of sleep (behavior) problems Teachers, school care coordinators and youth healthcare professionals in secondary education play a crucial role in identifying when students have sleep difficulties and to refer them to the appropriate healthcare. The aim of this CYB monitoring and screening tool is to provide guidance on recognizing sleep difficulties in students, initiating conversations with the student (and possibly their parents/guardians), and, if needed, referring the student to appropriate healthcare, in general the youth doctor or general practitioner	• Participation • Tailoring • Individualization • Using imagery • Elaboration • Consciousness raising • Planning coping responses • Self-monitoring of behavior • Goal setting • Planning coping responses	The tool includes: • A guide with detailed information on why sleep is so important for teenagers, how to recognize sleep deprivation, potential underlying causes, and recommended advice; • A screening questionnaire to help identify sleep difficulties in students; • A conversation tool to uncover the primary causes of sleep deprivation in the student. This tool includes a desk pad featuring a timeline and stickers pre-printed with sleep hygiene practices that teenagers can use to map out their afternoon, evening, night and morning routine

### The charge your Brainzzz program

The described steps resulted in the generation of eight program components that together form the Charge Your Brainzzz whole systems action program. These components are:

1) an educational component for first-year secondary school students comprising of four classes focusing on the themes sleep, stress and screen use; 2) a step-by-step guide for schools and Healthy School Advisors for formulating and implementing school sleep health policies; 3) a parent information evening to be held at school; 4) an online magazine for parents that serves as a reference guide which outlines how parents and caregivers can guide their teenagers toward better sleep; 5) the *Teen Sleep Check* serving as a tool for parents to find out how well their teenager is sleeping; 6) a *Sleep Guide* for adolescents and parents including practical tips for specific times of the day to ensure a better night’s sleep; 7) a tool for monitoring sleep health and early detection of sleep (behavior) problems; and 8) several implementation materials including a teachers’ manual for executing the educational program, a manual for Healthy School Advisors to implement the CYB program and an informative website. Table 4 presents an overview of all the Charge Your Brainzzz program components, used methods per intervention component and a description of the practical applications. Table 5 presents an overview of all implementation materials.

**Table 5:** Overview of the Charge Your Brainzzz implementation materials

8. Implementation materials	Practical applications/Tools
Teachers’ manual for educational program	This educational component was complemented with an extensive manual for teachers in order to facilitate its implementation. The teacher’s’ manual contains detailed instructions and explanations to help teachers effectively teach the material to students. It includes: • Lesson plans: Outlines of how to teach each lesson, including objectives, key concepts, and suggested timelines • Answers and solutions: Correct answers to exercises and problems in the textbook, often with explanations of how to arrive at those answers • Teaching strategies: Suggestions on how to present the material, engage students, and address different learning styles • Background information about sleep health to increase awareness about the importance of adolescent sleep health and its causes and consequences
Manual for Healthy School Advisors	This manual includes information about CYB (e.g., aim, content and planning of the program), information to convince schools to implement CYB within their program (e.g., information about the influence of sleep on school performances), and a list with techniques for schools to overcome barriers during implementation (e.g., time, money, support among school personnel). Additional materials included factsheets to spread among the schools and to use as a conversation tool with the school and a website to find information about the program
Charge Your Brainzzz website	An informative website (www.chargeyourbrainzzz.nl) designed to serve both recipients such as adolescents, and implementers, including education professionals (teachers, school leaders), care professionals (youth health care professionals, school care coordinators), and Healthy School Advisors. The site offers a wide range of resources and information focused on promoting better sleep. It provides educational content about the importance of sleep, practical tips for improving sleep health, detailed information about the CYB program components, and contact options to facilitate enrollment for implementation

**Fig. 3:**
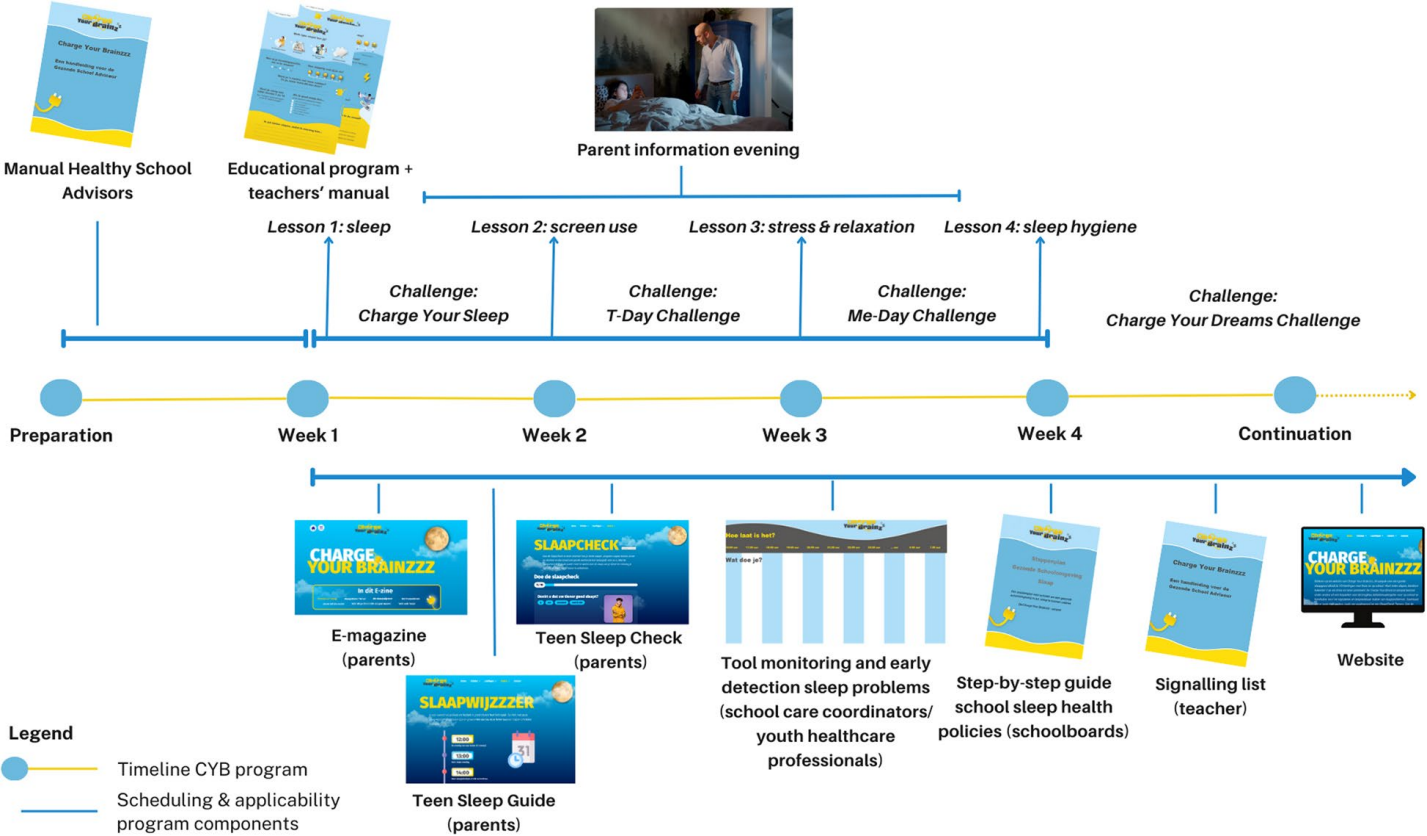
Charge Your Brainzzz program sequence

Figure 3 illustrates the total content, themes and sequence of the Charge Your Brainzzz program. Before the start, schools and/or their Healthy School Advisor receive the CYB manual instructing how to implement the CYB program (e.g., when to start, how and when to implement the school sleep health policies). After that, schools work with the educational program for approximately four weeks, during which about one lesson is taught each week, and commit themselves to a challenge in between lessons. During this period, the parent information evening is also held, and schools can utilize all other components of the CYB program. Aside from the educational program and the parent evening, the other program components can be used flexibly throughout the year.

## Discussion

This study aimed to describe the systematic development and details of the whole systems action program ‘Charge Your Brainzzz’ (CYB) designed to promote healthy sleep among Dutch adolescents aged 12–15. Building on the whole systems action *plan* of Heemskerk et al. [11], the program integrates theory- and evidence-based behavior change methods using the Intervention Mapping (IM) Protocol. This resulted in a comprehensive, co-created and theory- and evidence-based sleep intervention program tailored to pre-vocational education students. This work provides an empirical example of translating a whole systems action *plan* into a whole systems action *program* using behavior change techniques from Intervention Mapping, illustrating how a Systems Science approach and Intervention Mapping can complement each other in health promotion program development. Thereby, this paper serves as a guide for both systems scientists and intervention developers, demonstrating how these approaches can enhance and support each other in the development of health promotion programs. Additionally, it provides a detailed description of a whole systems action program promoting adolescent sleep health.

### Sleep health interventions for adolescents

Recent studies show a growing need to approach adolescent sleep health in a broader, more comprehensive manner to realize more impactful, lasting effects [6, 8, 9, 25]. Until now, most preventive sleep interventions have focused narrowly on a single aspect rather than taking a holistic approach to address the complex challenge of promoting adolescent sleep health. To date, such intervention programs usually consist of only an education program [6, 8, 26]. While these programs potentially reach large numbers of adolescents, and some have been shown to increase adolescents’ knowledge about sleep, none have shown meaningful, long-term effects on adolescent sleep health [8, 26–28].

The reasons for this could be multifaceted. One reason is that sleep education programs often lack a foundation in behavioral theory [6] which is key to achieving significant, sustained health behavior change [12, 29]. Another reason could be the lack of actor involvement in program development. Few adolescent sleep health promotion programs have thoroughly engaged stakeholders during program design and development [24]. This is likely to limit their appropriateness, acceptance, and feasibility among its recipients and implementers, thereby hampering successful implementation and effectiveness. Moreover, to our knowledge, no intervention program has integrated actions targeting adolescents with parallel efforts directed at *complementary stakeholders* – such as parents and education- and health professionals – who play a key role in influencing and shaping adolescent sleep health [30]. Finally, currently existing programs are usually not multi-component programs that also address the environmental and societal factors influencing sleep health alongside the personal behavioral determinants of adolescents [8, 25].

In developing the CYB program, we addressed the shortcomings of previous intervention programs by co-developing a whole systems action program targeting system dynamics and consisting of several intervention components beyond traditional educational components. These include, for example, a parent information session at school, an online Teen Sleep Check, a Sleep Guide for parents, a guide to include school sleep health policies and a tool for monitoring and early detection of sleep (behavior) problems for school care coordinators/youth healthcare professionals. The CYB program engages a variety of key stakeholders such as adolescents, parents, teachers, schoolboards, Healthy School Advisors, and school care coordinators/youth healthcare professionals, and it addresses not only personal behavior change but also broader changes in environmental and social determinants shaping sleep health. Furthermore, in order to facilitate the uptake of CYB, we ensured that CYB’s components were aligned with the Dutch Healthy School Approach framework [31]. In line with the evidence and call in a recent systematic review on school-based interventions for adolescent sleep health, which emphasizes the importance of developing whole-of-school approaches [8], CYB’s structure emerges as a promising response to this need, instilling considerable confidence it is potential effectiveness.

### Combining systems science with theory -and evidence-based behavior change methods

The development of the CYB program illustrates how Systems Science and Intervention Mapping can complement each other in designing theory – and evidence-based interventions, particularly for tackling complex or"wicked"problems. Using systems thinking as a basis for the development of health behavior change programs enhances the ability to account for intricate causal mechanisms and interactions among individual determinants, avoiding overly simplistic"solutions."This approach also improves the capacity to anticipate and manage unexpected or unintended outcomes [32]. For example, when delaying school starting times to align with adolescents’ biorhythm, it is essential to consider the potential impacts on school end times, after-school activities and evening homework. However, although these system dynamics illuminate the system mechanisms and actors involved in a particular health issue – in our case sleep health – they do not offer guidance on how to effectively and sustainably generate the necessary behavior changes among those actors within the identified system mechanisms. This is where systematic behavioral change methodologies such as Intervention Mapping prove invaluable. Such behavioral change approaches are needed to understand and change people’s behaviors within the system, given that human behavior drives every system dynamic (i.e., factor, connection, feedback loop, and underlying mechanism). For example, we have created the norm for adolescents to be 24/7 online available for school-related topics. In the absence of theoretically and empirically founded behavioral approaches, the translation of systems thinking into actual systems change becomes challenging.

The developers of the IM protocol recognize the complexity of health problems and encourage intervention designers to adapt the protocol to better capture this complexity [33]. To the best of the authors knowledge, this study is the first attempt to integrate Systems Science into the evidence-based practice of designing and implementing effective interventions. Using a Causal Loop Diagram as part of the ‘needs assessment’ – in which one aims to understand the health problem and use this information as the foundation for the program – proved to be an effective way to account for the complexity of health issue from the very beginning (System step 1, Fig. 1). This differs from the original more linear ‘logic model of the problem’ used as a first step in IM. Although more health problems are considered complex, the challenge thereafter remains: ‘what exactly should we do?’, and ‘how do we ensure that what we develop is effective?’. This study represents a step forward in addressing this gap: combining systems science with theory- and evidence-based methods to develop a more robust framework for tackling complex health issues.

### Strengths and limitations

Our study adopted a distinctive approach by combining participatory methods, systems thinking, and IM, with a primary focus on integrating theory- and evidence-based methods. This makes CYB the first comprehensive adolescent sleep health program built upon a robust theoretical and scientific foundation. By doing so, we advanced academic discourse on adolescent sleep health promotion as well as that on applying systems science and Intervention Mapping in complex health promotion program development.

Via our approach we were able to create a program that not only takes into account the complexity of the health problem targeting several system dynamics outcomes influencing sleep health, but also ensures that the components of the program are grounded in theory- and evidence. In addition, the stakeholder engagement (e.g., co-creation, interviews, co-designing) ensures that the program fits the lived experience of the target group of the intervention components (i.e., adolescents, parents and school care coordinators/youth healthcare professionals) and the implementers and embedders of the program (i.e., program owner, teachers and schoolboard and Healthy School Advisors). We recommend future research to integrate systems thinking and behavioral methodologies to enhance the robustness and effectiveness of interventions. Moreover, another notable strength of the program is its focus on pre-vocational secondary education students, a group that faces the most challenges with sleep compared to their peers in other educational tracks [3]. By focusing specifically on pre-vocational students, the program is both adapted to their health literacy and contributes to reducing health disparities. CYB thereby addresses a pressing need in Dutch public health [25, 34].

In addition, a system is dynamic, continuously adapting to changes within it [10]. Following program evaluation, it is expected that changes within the system will emerge. To address this, we recommend adopting an adapting, learning-oriented approach that allows the program to evolve in response to these observed changes. This approach involves ongoing monitoring and iterative revisions, enabling the program to integrate feedback, new evidence, and emerging needs over time. By remaining responsive and flexible, this strategy supports the program's long-term sustainability and enhances its capacity to deliver sustained outcomes by staying aligned with the system's evolving dynamics.

As shown in Fig. 1, the next steps, following the process outlined in the current article, will involve pilot testing the program to assess its feasibility and user appreciation (Step 5), followed by broader implementation in schools (Step 6). Subsequently, a comprehensive evaluation will be conducted using a control group design to assess both sleep-related outcomes and broader ripple effects on adolescents'well-being and functioning (Step 7). It is important to note, however, that these steps fall outside the scope of the present study, which focuses specifically on the systematic development of the intervention. Results from the pilot testing and subsequent evaluation will be reported in a separate publication.

Moreover, the program's current focus is on the school environment, addressing complex and interconnected factors within and across the school subsystem. However, the previously developed whole systems action plan [11] highlights that there are additional actions, settings, and stakeholders outside the school environment that play critical roles in improving adolescent sleep health. To maximize the program's impact, future iterations of the CYB program could be expanded to target other system dynamic outcomes, encompass other key settings and involve a broader array of stakeholders.

## Conclusion

This study introduces *Charge Your Brainzzz*, the first whole systems action program designed to promote adolescent sleep health. By targeting multiple actor groups and extending beyond traditional education focused solely on adolescents, the program addresses sleep health through a multifaceted lens. Using a Systems Science approach, the Intervention Mapping (IM) protocol, and stakeholder engagement, the program’s development incorporated the complexity of the health issue, the lived experiences of the target groups, and a solid theoretical foundation for behavior change, enhancing its potential effectiveness. This comprehensive approach tackles system dynamics across various interconnected subsystems, such as the school environment, digital landscape, mental wellbeing, family dynamics, and personal behavior, all influencing sleep health. Beyond presenting the program’s components, this study offers a replicable roadmap for addressing complex public health challenges, paving the way for innovative, system-oriented solutions in health promotion.

## Supplementary Information

Additional file 1. Interview structure for Healthy School Advisors

Additional file 2. Description of the co-creation structure

Additional file 3. Focus group structure youth healthcare professionals

Additional file 4. Example of how to form system dynamics outcomes for intervention program

Additional file 5. Performance Objectives and Change Objectives - School Environment

## Data Availability

The datasets generated and/or analysed are available on request from authors.

## Abbreviations

ASM: Action Scales Model
CYB: Charge Your Brainzzz
IM: Intervention Mapping

## Footnotes

1 The Dutch education system: Pupils entering secondary education in the Netherlands (usually around 12 years of age) are streamed according to aptitude into one of four forms of schooling: practical education (PrO), pre-vocational education (*vmbo*), senior general secondary education (*havo*), and pre-university education (*vwo*).

## Glossary

**Action Scales Model (ASM)**: Framework for understanding and addressing complex problems by distinguishing multiple levels (i.e., events, structures, goals and beliefs) to analyze a system and to intervene within a system to initiate systems change [23]
**Causal Loop Diagram (CLD)**: Visual representation of a system and its dynamics visualizing factors, their interrelationship and feedback loops [35].
**Charge Your Brainzzz (CYB)**: Whole systems action program in the Netherlands aiming to improve the system to promote the sleep health of Dutch adolescents aged 12–15 years.
**Intervention Mapping (IM)**: Systematic framework for developing theory- and evidence-based health promotion interventions and programs. It integrates theoretical knowledge, empirical evidence, and stakeholder input throughout the developmental process [12].
**Leverage points**: Modifiable points within a system. When these points are altered, this could lead to changes in the functioning, and thus the (health)outcome, of the system [10].
**Systems Science Approach**: A way of understanding and analyzing the world, health problem or other phenomenon that emphasizes the relationships and interactions between parts of a system rather than focusing on individual components in isolation [10].
**Whole Systems Approach (WSA)**: An approach to address complex problems by considering interconnected factors and relationships within an entire system to develop holistic and sustainable solutions [36].
**Whole Systems Actions Plan**: A plan/strategy that addresses complex problems by integrating and coordinating actions across all system levels and different subsystems and settings to achieve systemic, sustainable systems change [37].
**Whole Systems Action Program**: A program, grounded in theory and evidence-based behavior change methods, designed to address complex problems by integrating and coordinating actions across different system levels and its subsystems.
