# Supplementary material for: Charge Your Brainzzz: the systematic development of a whole systems action program promoting sleep health in adolescents
Source: BMC Public Health. 2025 Oct 17;25:3519. doi: 10.1186/s12889-025-23989-2 (PMC12535058; doi:10.1186/s12889-025-23989-2)
Supplement: Supplementary file 1 — Additional file 1. Interview structure for Healthy School Advisors [file 12889_2025_23989_MOESM1_ESM.doc]

**Additional File 1.** Interview Structure for Healthy School Advisors

**Participants:**

- Healthy School Advisors (HSAs)

**Objective:**

- Involve participants in the development process of the intervention (where are we now?).
- Identify the needs for tools related to adolescent sleep (ages 12-15).
- Gather feedback for the implementation of the intervention.

**Structure:**

1. **Introduction & Healthy School Advisor (HSA):**
   - Introduction of participant(s) and asking to clarify their roles within the Healthy School Approach.
   - Explanation of the objective: Gathering insights from HSAs on the specific needs and requirements to address the inadequate sleep health of adolescents.
2. **Role of Healthy School Advisors:**
   - What is the role of the HSA in schools? What are their tasks?
   - How are they involved in the development and/or implementation of interventions within schools?
3. **Introduction to Charge Your Brainzzz (CYB):**
   - Purpose and goals of CYB.
   - Overview of past efforts and key stakeholders involved.
   - Explaining the aim to align with the Healthy School framework.
4. **Healthy School Pillars:**
   - What existing components are in place to improve sleep health for adolescents within the Healthy School structure?
   - What intervention components would you like to see related to the Healthy School pillars (and why)?
     - Education
     - Environment
     - Policy
     - Monitoring
   - Do these components seem suitable for adolescents (ages 12-15)?
   - What criteria should these components meet to align with your approach?
   - What types of implementation materials would be helpful for you?
5. **Conclusion & Appreciation:**
   - Thank participants for their time and input.
