# Supplementary material for: Charge Your Brainzzz: the systematic development of a whole systems action program promoting sleep health in adolescents
Source: BMC Public Health. 2025 Oct 17;25:3519. doi: 10.1186/s12889-025-23989-2 (PMC12535058; doi:10.1186/s12889-025-23989-2)
Supplement: Supplementary file 2 — Additional file 2. Description of the co-creation structure [file 12889_2025_23989_MOESM2_ESM.doc]

**Additional file 2.** Description of co-creation session structure

| **Structure co-creation sessions** | **Aim** |
| --- | --- |
| *Prior: Pizza* | Eating pizza Short online questionnaire prompting participants to contemplate the topic independently, fostering personal opinions before starting a group discussion, and reducing social desirability bias. |
| 1. Introduction, acquaintance | - Eating pizza with the participants - Introducing the facilitators - Explaining the purpose of the co-creation session and informed consent information - Introducing the participants via a warming up exercise (e.g., “arrange yourselves in alphabetical order according to your first names” or “organize yourselves according to the duration of your sleep last night”). This ‘icebreaker exercise’ was aimed to create a comfortable atmosphere for participants. |
| 2. Identifying which learning methods young people find engaging and suitable for learning about sleep. | - Identifying how participations have encountered information about sleep and identifying gaps or patterns in their learning experiences:   - Participants engaged in the exercise: ‘glasses on, glasses off’, an activity designed to explore various ways participants have learned about sleep. Each participant is giving a pair of glasses. For every question: If their answer is yes, they put the glasses on. If their answer is no, they take the glasses off (or keep them off).   - Questions Asked:     - Have you ever learned or heard something about sleep at school?     - Have you ever learned about sleep from your parents or other people in your life?     - Have you ever learned or heard about sleep through TV, YouTube, Netflix, or social media?     - Have you ever looked up information about sleep on your own?   - For "Yes" Responses: The aim is to understand the impact of their prior learning experiences and what made those experiences effective or not *(e.g., “what did you learn/hear/look up/see?”, “was it helpful?”).*   - For "No" Responses: This helps uncover gaps in education or information and explores what might motivate participants to learn about sleep in the future *(e.g., “Would you have liked to learn about sleep at school, from your parents, or through Netflix/media?”, “why or why not?”, “What would you have liked to learn?”).* |
| 3. Identifying which themes from the lesson package are or are not engaging | - Before starting, a series of posters will be displayed around the room. Each poster will showcase a different example of an activity or learning method related to learning about sleep. These could include things like interactive games, videos, group discussions, or creative projects. The goal is to present a variety of approaches to learning about sleep. - Participants are invited to walk around the room and look at each poster. As they view the examples, they should think about whether the learning method would be engaging for them and effective in helping them understand sleep better. - Each participant will receive 3 green post-its.   - If they find a learning method they like, they will place a green post-it on the poster with that example.   - On the post-it, they should write why they think the method is good. This could include reasons like its fun, interactive, easy to understand, or it helps them retain information better. - Each participant will also receive 3 red post-its.   - If they find an example that they think is not effective or engaging, they will place a red post-it on that poster.   - On the red post-it, they should write why they think the method is not a good idea. This could be because the method feels boring, difficult to understand, or not relevant to them. - Facilitators choose which posters/learning methods will be discussed, for example, the posters with the most green post-its or the most red post-its. - Participants will walk around the room as a group, reviewing the green and red post-its. The group will talk about the reactions to each example, exploring why certain methods were liked or disliked. This discussion will give insight into what learning methods are most appealing and why |
| 4. Identifying how young people would design a lesson package (e.g., title, mascot, visuals, and overall theme) | - Individual drawing and group discussion:   E.g., Naming of the project:   - - Step 1: Brainstorming names: Each participant will receive an A4 sheet of paper. The task is to come up with fun and creative names for the lesson package (the overarching title of the project). Think about what would make the title catchy and engaging for your target audience.   - Step 2: Rotating papers: After a set amount of time (e.g., 5 minutes), everyone will stand up, leave their paper on the table, and move one seat to the right. Take the paper that’s now in front of you and add new name ideas or comments to the suggestions already written there. Continue brainstorming to further develop the names.   - Step 3: Selecting Favorites: After a few rounds of rotating papers, each participant will circle the names they find most appealing or creative. Once everyone has chosen their favorites, the group will gather to discuss the options and decide on the most popular or suitable name for the project. |
| 5. Closing | - The facilitators closed the session and asked questions such as: “what do you think of the results? Are there any questions or additional comments? How did you find the session? Is there anything you would like to share with the researchers?” - Explaining of what will be done with the input |
