# Supplementary material for: Charge Your Brainzzz: the systematic development of a whole systems action program promoting sleep health in adolescents
Source: BMC Public Health. 2025 Oct 17;25:3519. doi: 10.1186/s12889-025-23989-2 (PMC12535058; doi:10.1186/s12889-025-23989-2)
Supplement: Supplementary file 3 — Additional file 3. Focus group structure youth healthcare professionals [file 12889_2025_23989_MOESM3_ESM.doc]

**Additional File 3.** Focus group structure youth healthcare professionals

**Participants:**

- School care coordinator and youth healthcare professionals

**Objective:**

- Identify the needs for tools related to adolescent sleep (ages 12-15).

**Structure:**

1. **Introduction & Role of youth healthcare (Dutch: JGZ)**
   - Introduction of participants and their roles within JGZ.
   - Explanation of the objective: Gathering insights from JGZ to address identified needs.
2. **Role of JGZ in Schools:**
   - What is the role of JGZ in schools?
   - How do you communicate with parents and students?
3. **Introduction to Charge Your Brainzzz (CYB):**
   - Purpose and goals.
   - Overview of past efforts and key stakeholders involved.
   - Current developments focusing on prevention rather than sleep disorders.
4. **Practical Insights from JGZ:**
   - What signals are observed in practice?
   - What are your specific needs regarding sleep and adolescents? (ages 12-15).
   - What is already there?
   - Current approaches:
     - Is sleep systematically addressed within this age group?
   - Identified gaps:
     - Needs for parent communication?
     - Needs for engagement with adolescents/students?

**Prompt Questions:**

- Is there a need for a visual discussion aid ("praatplaat")?
  - Should it differ for parents and teenagers?
- What should the visual aid look like?
  - Online or physical format?
  - Placemat or brochure?
  - Illustrations: Animations or photographs?
