# Supplementary material for: Charge Your Brainzzz: the systematic development of a whole systems action program promoting sleep health in adolescents
Source: BMC Public Health. 2025 Oct 17;25:3519. doi: 10.1186/s12889-025-23989-2 (PMC12535058; doi:10.1186/s12889-025-23989-2)
Supplement: Supplementary file 4 — Additional file 4. Example of how to form system dynamics outcomes for intervention program [file 12889_2025_23989_MOESM4_ESM.pdf]

Additional file 4. Example of how to form system dynamics outcomes for intervention program

In the research conducted by Heemskerk et al. (15), a comprehensive Causal Loop Diagram (CLD) was developed to integrate all relevant determinants of adolescent sleep health, underlying system dynamics (e.g., connections, feedback loops, and subsystems), and potential leverage points for systems change. The system was analyzed across various levels using the Action Scales Model to identify leverage points at each level.

Following the framework of Nobles et al. (1), the Action Scales Model identifies several system levels:

- **Event level:** Issues arising from how the system functions.
- **Structure level:** The system's organization, which drives events and patterns.
- **Goal level:** The targets the system actively works to achieve.
- **Belief level:** The deeply held beliefs of system leaders regarding priorities and strategies.

Leverage points at the event and structure levels were often visible within the CLD, while those at the goal and belief levels were analyzed by the research team. Identified leverage points included elements that drive feedback loops, are part of feedback loops, have high connectivity (e.g., numerous incoming and outgoing connections), or exhibit strong causal relationships with sleep dimensions.

This additional file illustrates the process of transitioning from the CLD and system dynamics analysis (see figure) to system dynamics outcomes (see table) for the subsystem "school environment". Key dynamics for other subsystems—such as mental well-being, digital environment, family and home environment, health behaviors and leisure activities, and the personal system—are not included in this example. The identified feedback loops (R1 a,b,c, R2, R3 and B1 within the figure correspond with the numbers in the first column in the table.

Figure Additional file 4. Causal Loop Diagram representing system dynamics related to the school environment

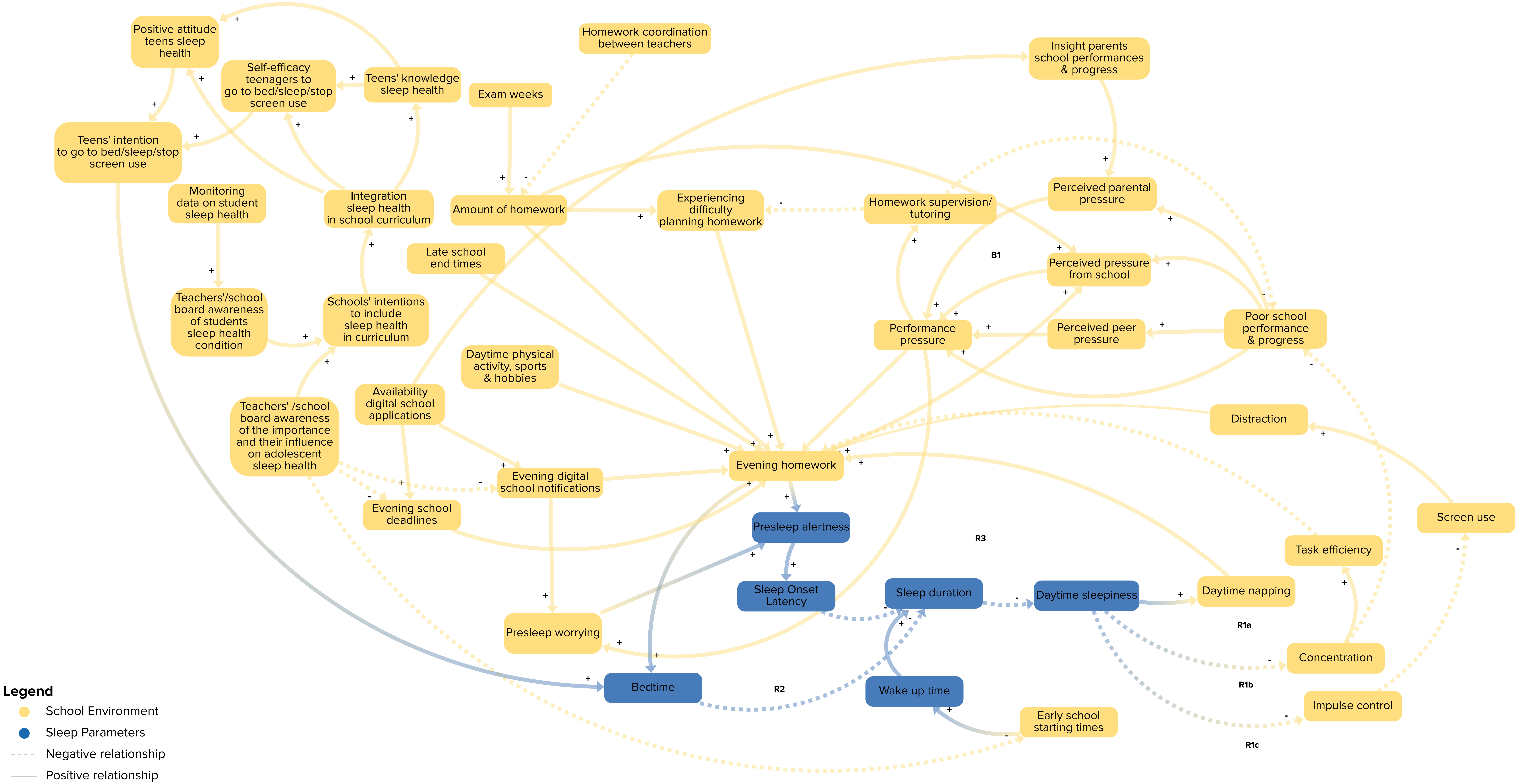

Positive attitude teens sleep health
Self-efficacy teenagers to go to bed/sleep/stop screen use
Teens' knowledge sleep health
Homework coordination between teachers
Insight parents school performances & progress
Exam weeks
Teens' intention to go to bed/sleep/stop screen use
Monitoring data on student sleep health
Integration sleep health in school curriculum
Amount of homework
Experiencing difficulty planning homework
Homework supervision/ tutoring
Perceived parental pressure
Late school end times
B1
Perceived pressure from school
Teachers'/school board awareness of students sleep health condition
Schools' intentions to include sleep health in curriculum
Performance pressure
Perceived peer pressure
Poor school performance & progress
Daytime physical activity, sports & hobbies
Availability digital school applications
Teachers' /school board awareness of the importance and their influence on adolescent sleep health
Distraction
Evening homework
Evening digital school notifications
Evening school deadlines
Presleep alertness
R3
Screen use
Task efficiency
Sleep Onset Latency
Sleep duration
Daytime sleepiness
Daytime napping
R1a
Presleep worrying
Concentration
R1b
Bedtime
R2
Wake up time
Impulse control
Early school starting times
R1c
Legend
School Environment
Sleep Parameters
Negative relationship
Positive relationship

Table Additional file 4. Feedback loops, leverage points and system dynamics outcomes subsystem 'school environment'

Feedback loops as identified in Heemskerk et al. (15): School environment	Previous identified leverage points on the Event, Structure, Goal and Believe level of the system as identified in Heemskerk et al. (15):	System dynamics outcomes prioritized for CYB
<u>**Evening homework**</u><br>**R1:** Evening homework ↑ - presleep alertness ↑ - Sleep onset latency ↑ - sleep duration ↓ - daytime sleepiness ↑ - (see a, b, c).<br><br>**R2:** Evening homework ↑ - (postponing) bedtime ↑ - sleep duration ↓ - daytime sleepiness ↑ - (see a, b, c).<br><br>*Consequences of daytime sleepiness:*<br>a. Daytime napping ↑ - evening homework ↑<br>b. Concentration ↓ - task efficiency ↓ - evening homework ↑<br>c. Impulse control ↓ - screen use ↑ - distraction ↑ /task efficiency ↓ - evening homework ↑<br><br><u>**Performance pressure from school**</u><br>**B1:** performance pressure through self-inducing, peers, school and parents ↑ - homework supervision/tutoring ↑ - poor school performance ↓- performance pressure through self-inducing, peers, school and parents ↓<br><br>**R3** - performance pressure through self-inducing, peers, school and parents ↑ - evening homework ↑ - …<br>a. …presleep alertness ↑ - Sleep onset latency ↑ - …	**Event:**<br>● Evening homework<br>● Difficulties students in planning their homework<br>● Perceived pressure from school<br><br>**Structure:**<br>● Misalignment school schedules with adolescents' biorhythm (e.g. starting- and end times, break times, exam times)<br>● Lack of homework coordination between teachers about when and how much homework they give<br>● Late-night school deadlines<br>● Digital communication about homework from schools in the evening<br>● Sleep health, and relaxing sleep hygiene practices are not included as part of the school curriculum<br>● Limited monitoring data on student sleep health<br><br>**Goals:**<br>● Providing students with optimal cognitive, educational outcomes (not promoting adolescent (sleep) health)<br><br>**Beliefs:**	**Event:**<br>● Adolescents doing evening homework<br>● Adolescents experience difficulties planning their homework<br>● Adolescents perceive performance pressure from school<br><br>**Structure:**<br>● School schedules are not aligned with adolescents' biorhythm (e.g. starting- and end times, breaks)<br>● Homework is not coordinated between teachers about when and how much homework they give<br>● Late-night school deadlines are set<br>● Schools digitally communicate about e.g., homework in the evening<br>● Sleep health and relaxing sleep hygiene practices are not part of the school curriculum<br><br>**Goals:**<br>● School provides students with optimal cognitive, educational outcomes and have no focus on promoting adolescent (sleep) health)<br><br>**Beliefs:**<br>● Schools are there to facilitate learning and getting good grades

b. …bedtime ↑ -…<br><br>…sleep duration ↓ - daytime sleepiness ↑ - concentration ↓ - poor school performances & progress ↑- performance pressure through self-inducing, peers, school and parents ↑<br><br>**<u>School start times</u>**<br>No feedback loops identified	● Schools are there to facilitate learning and getting good grades<br>● Lack of teachers'/school board awareness of students sleep health (or mental health) condition<br>● Lack of a belief or awareness that schools have a large responsibility in stimulating sleep health	● Teachers'/school boards have a lack of awareness of students sleep health (or mental health) condition<br>● Schools have a lack of belief or awareness that they have a large responsibility in stimulating adolescent sleep health

R: Reinforcing feedback loop: indicating that an increase in variable A leads to its further increases.
B: Balancing feedback loop: indicating that an increase in a variable ensures its future decrease, thereby creating a balance.
↑: Increase in factor
↓: Decrease in factor
