# Supplementary material for: Charge Your Brainzzz: the systematic development of a whole systems action program promoting sleep health in adolescents
Source: BMC Public Health. 2025 Oct 17;25:3519. doi: 10.1186/s12889-025-23989-2 (PMC12535058; doi:10.1186/s12889-025-23989-2)
Supplement: Supplementary file 5 — Additional file 5. Performance Objectives and Change Objectives - School Environment [file 12889_2025_23989_MOESM5_ESM.doc]

**Additional File 5.** Performance Objectives and Change Objectives - School Environment

| **School environment** | **Change objectives** | | | | | |
| --- | --- | --- | --- | --- | --- | --- |
| **Performance Objectives** | **Knowledge** | **Awareness** | **Attitude/beliefs** | **Self-efficacy/barriers** | **Subjective norms** | **Skills** |
| **Adolescents** plan (with help of their **parents**) their daily activities in such a way to prevent homework in the evening | Adolescents know and recognize the negative influences of evening homework on their sleep  Parents know the negative influences of evening homework on the sleep of adolescents | Adolescents become aware of their activities during the day and opportunities for better planning  Parents become aware of the perceived difficulties of adolescents with planning daily activities (including homework) | N/A  Parents belief that adolescents at this age range cannot always take their own responsibility and still need a guiding role from their parents | Adolescents identify barriers for not finishing homework in the afternoon  Express confidence in planning their daily activities in such a way to prevent homework in the evening  Parents feel confident in providing help to their child with planning daily activities (including homework) | N/A  Parents recognize that other parents also provide help with planning daily activities with their child | Demonstrate a plan of how to make an efficient planning of daily activities preventing evening homework  N/A |
| **Teachers** avoid assigning homework for the following day  **Teachers** communicate homework details in time, at the latest at 4:00 PM | Teachers know and recognize the negative effects of evening homework on adolescents’ sleep health | Become aware of their influence regarding assigning/communicating homework not on time on adolescent sleep | Believe that that their students have the right to leisure time after school hours to keep a healthy school-private life balance and protect their mental wellbeing | Identify barriers for not assigning/communicating homework for the next day/on time  Express confidence in not assigning/communicating homework for the next day/on time | N/A | N/A |
| **Schools** have a policy stating to assign little to no homework for the next day  **Schools** create a structure to provide support and guidance to adolescents in completing homework on time  **Schools** aim to provide support and guidance to adolescents in completing homework on time  **Schools** become aware that their students have the right to leisure time after school hours to keep a healthy school-private life balance and protect their mental wellbeing | Schools know and recognize the benefits of diminishing evening homework on adolescents’ sleep health and providing support and guidance in completing homework on time | Become aware of their influence regarding assigning/communicating homework not on time and providing support and guidance in completing homework on time on adolescent sleep | Believe that that their students have the right to leisure time after school hours to keep a healthy school-private life balance and protect their mental wellbeing | Identify barriers for not developing and implementing a policy regarding not assigning/communicating homework for the next day/on time  Identify barriers for not providing guidance and support for students to complete homework on time  Express confidence in developing and implementing a policy/agreement regarding not assigning/communicating homework for the next day/on time and setting up a structure to support and guide students in completing homework on time | Recognize that other schools also aim to prevent evening homework and provide support and guidance for students to complete homework on time | Demonstrate the ability to implement a policy/agreement that little to no homework is assigned for the next day  Demonstrate the ability to provide support and guidance in completing homework on time |
| **Teachers** avoid setting late-night school deadlines | Teachers know and recognize the negative effects of setting late-night school deadlines on adolescents’ sleep health | Become aware of their influence regarding setting late-night school deadlines on adolescent sleep | Believe that that their students have the right to leisure time after school hours to keep a healthy school-private life balance and protect their mental wellbeing | Identify barriers for not setting late-night school deadlines  Express confidence in not setting late-night school deadlines | N/A | N/A |
| **Schools** implement a policy that restricts late-night school deadlines  **Schools** become aware that late-night school deadlines damage students’ sleep health, which in turn is crucial for their cognitive development, mental wellbeing and physical health as well as damaging their right to leisure time after school hours to keep a healthy school-private life balance and protect their mental wellbeing | Schools know and recognize the benefits of restricting late-night school deadlines on adolescents’ sleep health | Become aware of their influence regarding late-night school deadlines on adolescent sleep | Believe that late-night school deadlines damage student sleep health, which in turn is crucial for their cognitive development, mental wellbeing and physical health as well as damaging their right to leisure time after school hours to keep a healthy school-private life balance and protect their mental wellbeing  Feel positive about restricting late-night school deadlines | Identify barriers for restricting late-night school deadlines  Express confidence in developing and implementing a policy/agreement that restricts late-night school deadlines | Recognize that other schools also aim to restrict late-night school deadlines | Demonstrate the ability to implement a policy/agreement that restricts late-night school deadlines |
| **Teachers** coordinate homework assignments and exams with other subjects to prevent workload peaks | Teachers know and recognize the negative effects of workload peaks on adolescents’ sleep health | Become aware of their influence regarding coordinating homework and exams and workload peaks on adolescent sleep | Feel positive about coordinating homework assignments and exams with other subjects to prevent workload peaks, to keep a healthy school-private life balance and protect students’ mental wellbeing | Identify barriers for not coordinating homework assignments and exams with other subjects  Express confidence in coordinating homework assignments and exams with other subjects | N/A | N/A |
| **Schools** schedule no exam weeks, but rather distribute exams evenly throughout each trimester to prevent peaks in workload and subsequent stress with students  **Schools** utilize one digital platform for communication and sharing homework assignments with adolescents to prevent unexpected workload peaks | Schools know and recognize the benefits of restricting exam weeks and using one digital platform for communication with students on adolescents’ sleep health | Become aware of their influence regarding exam weeks and communication structures on adolescent sleep | Feel positive about distributing exams evenly throughout each semester and utilizing one digital platform for communication | Identify barriers for restricting exam weeks and using one digital platform to communicate with students  Express confidence in spreading exams evenly throughout each trimester and using one digital platform to communicate with students | Recognize that other schools also aim to restrict exam weeks to prevent stress with students | Demonstrate the ability to change exam schedules and communication structures with students |
| **Schools** implement a policy that states that they align school schedules with adolescents’ biorhythm (e.g., start times and end times, breaks)  **Schools** implement a policy that tests or examinations do not take place before 11:00 AM  **Schools** aim to align school schedules with students’ biorhythm    **Schools** become aware of the impact of school start time on sleep and cognitive development, and the importance of better aligning school start times with adolescent biorhythm | Schools know and recognize the benefits of aligning school schedules with adolescents’ biorhythm  Recognize that later school start times are not associated with later school end times | Become aware of their influence regarding school schedules on adolescent sleep  Recognize the need for later school starting times and later timing of examinations to improve adolescent sleep health | Believe that sleep health is crucial for the mental health and cognitive development of adolescents  Believe that later school start times and later timing of examinations align better with students’ biorhythm and is beneficial for improving adolescent sleep health, mental wellbeing and school performances  Feel positive about delaying school start times and examinations | Identify barriers for delaying school start times and examinations  Express confidence in developing and implementing later school start times and later timing of examinations | Recognize that other schools also aim to improve and protect adolescent sleep health | Demonstrate the ability to implement later school start times and later timing of examinations |
| **Teachers** do not specify the exact day and time that they will publish students' test scores and grade  **Teachers** do not digitally communicate in the evening  **Teachers** become aware about the effect of evening communication and specifying the exact day and timing of grade publication on sleep health to strengthen the belief that this anticipation causes stress and in turn affects sleep health | Teachers know and recognize the negative effects of communicating school related topics in the evening and specifying the exact day and time in the evening that they will publish students’ test scores adolescents’ sleep health | Become aware of their influence regarding communicating school related topics in the evening and specifying the exact day and time in the evening on adolescent sleep | Believe that communicating school related topics in the evening and specifying the exact day and timing of grade publication causes stress and in turn negatively affects sleep health | Identify barriers for not communicating in the evening and specifying the exact day and timing of grade publication  Express confidence in not communicating in the evening and specifying the exact day and timing of grade publication | N/A | N/A |
| **Schools** only use digital (mobile) applications that allow for sending students messages at preset times  **Schools** implement a policy that prohibits teachers from specifying when test grades will be published    **Schools** set their digital (mobile) applications to limit the accessibility of students to the online grading and test scores system between 8:00 PM and 8:00 AM    **Schools** implement policies that prevent sending students notifications as well as to prevent them from accessing the online test scores and grading system between 8:00 PM and 8:00 AM  **Schools** become aware that evening notifications (e.g. communicating test results) causes students stress that in turn negatively affects their sleep health  **Schools** become aware that their students have the right to leisure time after school hours to keep a healthy school-private life balance and protect their mental wellbeing | Schools know and recognize the negative effects of 24/7 accessibility of students to online grading and test scores system on evening stress and sleep health | Become aware of their influence regarding 24/7 availability of digital school applications on adolescent stress levels and sleep health  Recognize the need for limiting accessibility of students to the online grading and test scores system between 8:00 PM and 8:00 AM | Believe that evening notifications (e.g. communicating test results) causes students stress that in turn negatively affects their sleep health (Belief)  Feel positive about limiting accessibility of students to the online grading and test scores system between 8:00 PM and 8:00 AM | Identify barriers for limiting accessibility of students to the online grading and test scores system between 8:00 PM and 8:00 AM  Express confidence in limiting accessibility of students to the online grading and test scores system between 8:00 PM and 8:00 AM | Recognize that other schools also limit accessibility of students to the online grading and test scores system to improve and protect adolescent sleep health | Demonstrate the ability to limit accessibility of students to the online grading and test scores system between 8:00 PM and 8:00 AM |
| **Adolescents** learn about sleep health in school with the aim to stimulate their healthy sleep habits | Describe and recognize the importance of sleep hygiene practices and adequate sleep health  Know the recommended amount of sleep for their age  Being aware of the negative effects of inadequate sleep health  Describe and recognize the advantages of having a relaxing bedtime routine  List tips to improve their sleep hygiene practices | Become aware of their own healthy and unhealthy sleep hygiene practices | Feel positive about improving their sleep health | Identify the source of their unhealthy sleep hygiene practices  Express confidence in improving their sleep hygiene practices and sleep health |  | Demonstrate a plan of how to improve their sleep hygiene practices and sleep health |
| **Schools** integrate sleep health intervention efforts into their curriculum  **Schools** actively protect and stimulate the sleep health of their students  **Schools** become aware of their students’ sleep health condition  **Schools** monitor students’ sleep health condition regularly  **Schools** prioritize overall well-being alongside academic achievement  **Schools** become aware that adolescent sleep health is crucial to healthy adolescent development, cognitive functioning and learning | Schools know and recognize the benefits of improving adolescent sleep health on cognitive development, mental wellbeing, physical health and school performances  Know where to find help for integrating sleep health interventions into their curriculum | Become aware of their influence on adolescent sleep health  Recognize the need for integrating sleep health interventions within the school curriculum limiting accessibility of | Believe that sleep health intervention efforts at school is crucial for their cognitive development, mental wellbeing, physical health and cognitive functioning  Feel positive about implementing sleep health intervention efforts in their curriculum | Identify barriers for implementing sleep health intervention efforts within the school curriculum  Express confidence in implementing sleep health intervention efforts within the school curriculum | Recognize that other schools also implement sleep health intervention efforts within the school curriculum | Demonstrate the ability to find ways to implement sleep health intervention efforts within the school curriculum |
